# FroHyNet: frozen temporal priors and trainable hypergraph fusion for brain time-series classification

**DOI:** 10.3389/fmed.2026.1966334

**Published:** 2026-10-07

**Authors:** Mingyao Gao, Ling He, Wen Li, Yajing Meng, Qing Li, Changjian Qiu, Jing Zhang

**Affiliations:** 1 College of Biomedical Engineering, Sichuan University, Chengdu, China; 2 Mental Health Center, West China Hospital, Sichuan University and Sichuan Clinical Medical Research Center for Mental Disorders, Chengdu, China

**Keywords:** brain time-series classification, fMRI, fNIRS, frozen temporal encoder, hypergraph neural network, temporal foundation model

## Abstract

**Background:**

Functional brain time-series classification is constrained by small clinical cohorts. Handcrafted features are sample-efficient but discard temporal detail; end-to-end networks must estimate many temporal parameters from limited labels; and frozen time-series foundation models reduce that estimation burden but typically encode each channel independently, leaving coordinated activity across brain regions unmodeled.

**Methods:**

We introduce the Frozen Temporal-Hypergraph Network (FroHyNet), an asymmetric parallel framework that preserves channel-wise temporal representations from a frozen MantisV2 encoder while a trainable spatio-temporal hypergraph branch learns topology-aware spatial corrections. Bottleneck-mix Top-K (BM-TopK) then residual-injects these corrections only along selected channel and latent-feature axes, treating the spatial stream as a selective update rather than a dense overwrite of the temporal representation. We conduct five-fold cross-validation across two functional near-infrared spectroscopy (fNIRS) and three functional magnetic resonance imaging (fMRI) datasets.

**Results:**

FroHyNet achieves the best average accuracy and area under the receiver-operating-characteristic curve (AUC) among methods evaluated on all five datasets, while keeping the trainable-parameter count well below those of from-scratch temporal networks. Gains are concentrated on fNIRS, with more modest improvements on the smaller fMRI cohorts. Branch ablations indicate dataset-dependent complementarity between the two streams, and fusion ablations on HC-D favor BM-TopK over the evaluated dense and recurrent alternatives.

## 1 Introduction

Functional near-infrared spectroscopy (fNIRS) and functional magnetic resonance imaging (fMRI) measure hemodynamic activity as multichannel time series (1). Through neurovascular coupling, local blood-oxygenation changes evolve over time and form coordinated patterns across distributed brain regions, providing informative cues for clinical and behavioral classification (1, 2). Reliable learning remains difficult, however, because labeled cohorts are costly to assemble and recordings exhibit substantial inter-individual variability, physiological noise, and scanner- or protocol-specific shifts (3).

Current approaches trade representation capacity against sample efficiency. Handcrafted pipelines commonly summarize signals as statistics or connectivity matrices (4, 5); although sample-efficient, this compression removes temporal detail. End-to-end temporal networks retain the raw trajectory (6, 7), yet their many trainable parameters can yield unstable generalization estimates in small clinical cohorts (8). Graph and hypergraph models are designed to encode pairwise and higher-order coordination among channels (9, 10), but typically fold the temporal signal into connectivity construction and do not retain a separate temporal stream. Frozen time-series foundation models reuse pretrained temporal representations while limiting the parameters estimated from downstream labels (11–13). Many such encoders process channels independently and therefore do not explicitly model coordinated activity among brain regions (11–13).

We therefore keep pretrained temporal features in one branch and learn pairwise and higher-order spatial structure in a second, parallel branch. We call this model the Frozen Temporal–Hypergraph Network (FroHyNet). A frozen MantisV2 encoder (14) preserves channel-wise temporal representations, while a trainable spatio-temporal hypergraph neural network (ST-HGNN) branch learns pairwise and sample-adaptive higher-order spatial relations. Whereas existing brain-network methods fold temporal information into connectivity construction or windowed graph sequences (5, 15–17), FroHyNet retains the frozen temporal stream as the residual carrier while learning graph and hypergraph corrections in a separate parallel branch. Bottleneck-mix Top-K (BM-TopK) jointly scores the two streams and injects spatial corrections only along selected channel and latent-feature axes, unlike dense fusion that mixes all channels and features. We evaluate this design against handcrafted, end-to-end temporal, frozen-foundation, domain-specific, and graph or spatio-temporal baselines on five fNIRS and fMRI datasets.

The main contributions are:

- *Asymmetric parallel temporal–spatial architecture:* FroHyNet preserves channel-wise temporal representations from frozen MantisV2 (14) while learning topology-aware spatial corrections through a trainable dynamic-hypergraph branch (18). Encoding the two views independently prevents graph processing from replacing the temporal stream before fusion.
- *BM-TopK sparse residual fusion:* A bottleneck-mix dual-axis Top-K gate provides sparse residual injection along selected channel and latent-feature axes, $Z_{f}=Z_{t}+(g_{c}⊗g_{d})⊙Z_{s}$. This design restricts dense spatial overwrite relative to concatenation or full attention. Selected coordinates are reported as model-internal diagnostics rather than as direct physiological markers.
- *Five-dataset results:* Across five fNIRS and fMRI datasets under five-fold cross-validation, FroHyNet attains 74.13% mean accuracy (ACC) and 74.75% mean area under the receiver-operating-characteristic curve (AUC) with 0.118M trainable parameters, providing a favorable accuracy–trainable-parameter trade-off against from-scratch temporal models. The aggregate gain is driven mainly by the two fNIRS datasets (96.17% mean ACC); the three smaller fMRI cohorts reach 59.44% mean ACC. Branch ablations indicate dataset-dependent parallel complementarity, while fusion ablations on HC-D favor BM-TopK over the evaluated dense and recurrent alternatives.

## 2 Related work

### 2.1 Temporal modeling for brain time series

Learning discriminative representations from functional brain time series has long been constrained by scarce labels and noisy multichannel recordings. Early clinical pipelines, especially in fNIRS, summarize each channel with temporal statistics of oxygenated hemoglobin, spectral energy, or functional-connectivity descriptors (5, 19). These handcrafted features are interpretable and parameter-efficient, which makes them attractive for small cohorts. We therefore retain handcrafted multilayer perceptron (MLP) and graph convolutional network (GCN) pipelines as small-sample controls. Their predefined compression discards much of the raw temporal trajectory that may distinguish clinical or behavioral states. End-to-end temporal networks reverse this trade-off by learning directly from the raw signal. Architectures such as ResNet-1D, a one-dimensional adaptation of ResNet (20), iTransformer (6), Medformer (7), and Leddam (21) can recover fine temporal structure when data are abundant. Yet every temporal weight must still be estimated from the target cohort, which can yield unstable generalization estimates under limited clinical labels (8), and early channel concatenation can propagate noise across the network. Frozen temporal foundation models, including NuTime (13), MantisV2 (14), TimesFM (22), Mantis-8M (11), PatchTST (23), and MOMENT (12), reuse pretrained sequence representations. They optimize only a lightweight downstream head and thereby reduce the number of parameters fitted to cohort labels. Several public temporal foundation models encode channels as separate sequences (11–13) and therefore leave multi-region interactions largely implicit. These methods either compress temporal detail for robustness, train high-capacity temporal models from limited labels, or freeze strong temporal priors without modeling brain-network topology.

### 2.2 Domain-specific brain time-series models

Domain-specific models add neuroimaging assumptions to temporal architectures. TeCh (24) and DeCI (25) incorporate assumptions tailored to brain-disorder series. In parallel, fNIRS-T and its pretrained variant fNIRS-PreT (26), LoBrAFrame (27), and MaNDiNeT (28) specialize in fNIRS decoding. Relative to generic temporal learners, these models strengthen neuroimaging inductive bias. They still primarily address temporal encoding, not higher-order spatial structure among brain regions.

### 2.3 Graph and hypergraph models of brain connectivity

A separate literature focuses on spatial coordination among brain regions. Graph neural networks represent pairwise coupling through message passing on adjacency graphs (10, 29). Hypergraph models extend this idea to groupwise co-activation among multiple regions (9, 30). Dynamic mixtures of graph and hypergraph messages have also been studied in forecasting (18). In neuroimaging, models such as STAGIN (15), MDGL (17), SimMVF (16), BrainOOD (31), and MVHO (5) exploit connectivity for classification or representation learning. These methods organize temporal and spatial information in different ways; none follows a single temporal-then-spatial pattern. STAGIN and MDGL construct sequences of windowed functional-connectivity graphs, encode each graph spatially, and then aggregate the resulting graph representations over time (15, 17). SimMVF and MVHO instead derive multi-view or higher-order functional networks from the time series before network fusion or classification (5, 16), while BrainOOD learns feature and structure selection directly on input brain networks (31). Across these approaches, temporal information enters the graph pathway through connectivity construction or graph snapshots; a separate frozen channel-wise temporal stream is not retained as a residual carrier.

### 2.4 Fusion of temporal and spatial streams

How the temporal and spatial streams are combined also matters. Common fusion operators include concatenation, adaptive gating, recurrent mixing, cross-attention (32), and token aggregation–redistribution. These mechanisms usually update all channels or latent features at once. When spatial estimates are unreliable, dense mixing can overwrite temporal cues with inaccurate spatial corrections.

FroHyNet retains frozen channel-wise temporal priors from MantisV2 and learns pairwise and higher-order spatial structure with a trainable ST-HGNN branch in parallel, not in series. BM-TopK then residual-injects spatial corrections only along selected channel and feature axes. We therefore compare against handcrafted, end-to-end, frozen-foundation, domain-specific, and graph or spatio-temporal baselines. We also ablate both branch layout and fusion operators under matched conditions.

## 3 Methodology

### 3.1 Problem definition and architecture overview

Let $x\in R^{B\times C\times T}$ denote a batch of functional brain time series, where B is the batch size, C is the number of channels or brain regions, and T is the sequence length. The task is to predict a binary clinical or behavioral label y. When available, the model also receives an adjacency matrix $A\in R^{C\times C}$ and a hypergraph incidence matrix $H\in R^{C\times E}$, where E is the number of hyperedges.

FroHyNet uses an asymmetric parallel temporal–spatial architecture (Figure 1; Table 1). The two branches process the same input x and produce aligned node-level representations: a frozen MantisV2 temporal branch yields $Z_{t}\in R^{B\times C\times D}$, and a trainable ST-HGNN spatial branch yields topology-aware $Z_{s}\in R^{B\times C\times D}$. BM-TopK then applies dual-axis Top-K gating to residual-inject selected spatial corrections, forming $Z_{f}$, which is pooled and classified. The frozen branch preserves pretrained temporal priors, whereas the trainable branch estimates task-specific network structure. Downstream optimization updates only the spatial branch, fusion module, optional alignment projections, and classifier.

**Figure 1:**
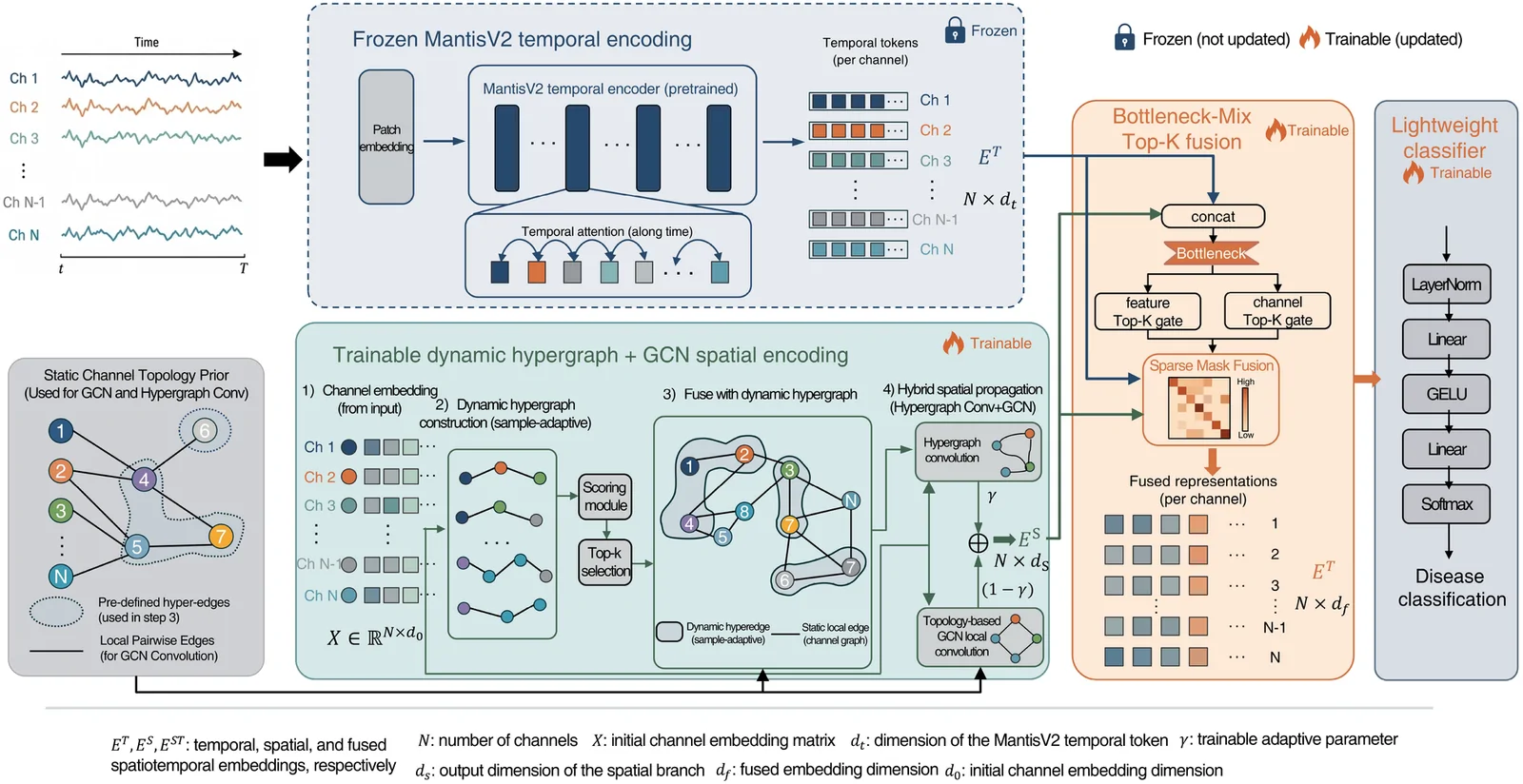
Overview of FroHyNet. A frozen MantisV2 temporal branch and a trainable dynamic-hypergraph spatial branch process the same multichannel input in parallel; bottleneck-mix Top-K (BM-TopK) fuses the embeddings by bottleneck-mix Top-K gating before a lightweight classifier. Locked modules stay frozen during downstream training.

**Table 1:** FroHyNet pipeline stages, modules, and their outputs.

Stage	Module	Output/Role
Input	Raw time series + optional A, H	Shared input to both branches
Temporal	Frozen MantisV2 (+ optional linear align)	$Z_{t}$: temporal embedding per node
Spatial	Compressor + hypergraph convolution (HGConv) / graph convolution (GCN) blocks	$Z_{s}$: topology-aware embedding per node
Fusion	Bottleneck-mix Top-K (BM-TopK)	$Z_{f}=Z_{t}+(g_{c}⊗g_{d})⊙Z_{s}$
Prediction	Pool + LayerNorm + Dropout + Linear	Class logits $\hat{y}$

Frozen MantisV2 is the temporal encoder; HGConv/GCN denotes hypergraph convolution and graph convolution in the spatial branch; BM-TopK is bottleneck-mix Top-K residual fusion. A and H are the optional adjacency and hypergraph incidence matrices.

### 3.2 Parallel temporal–spatial branches

The temporal branch must retain sequence information without estimating a high-capacity encoder from the target cohort. We therefore apply MantisV2 (14) independently to each channel and freeze its parameters during downstream training. The encoder produces node-level representations $Z_{t}\in R^{B\times C\times D}$, followed by an optional linear projection when dimensional alignment is required. This branch acts as the residual carrier throughout fusion, so the spatial stream modifies rather than replaces the pretrained temporal representation.

The temporal branch does not explicitly represent coordination among regions. The spatial branch therefore combines pairwise graph convolution with hypergraph convolution over multi-region groups. Dynamic hyperedges are constructed from the top-k cosine similarities among node representations. Following (18), a learnable coefficient γ mixes graph and hypergraph messages, with the operators adapted to brain tensors of shape [B,C,D].

The spatial branch is fully trainable. A lightweight temporal compressor first maps each channel to an initial node state. Stacked ST-HGNN blocks then propagate information through the dynamic hypergraph and, when provided, the pairwise graph. When an adjacency matrix A is provided, as for fMRI, pairwise graph convolution uses a training-split-only functional-connectivity graph, and sample-adaptive hyperedges are constructed from the learned node representations; no external anatomical incidence matrix H is required. For fNIRS, neither external A nor H is used, and the channel-level dynamic hypergraph is constructed from the data. The branch returns $Z_{s}\in R^{B\times C\times D}$, aligned with the temporal representation.

### 3.3 BM-TopK fusion and classification

Dense fusion allows every spatial estimate to alter the pretrained temporal representation. BM-TopK instead treats the spatial stream as a sparse residual correction. Given aligned representations $Z_{t},Z_{s}\in R^{B\times C\times D}$, it first forms a joint scoring view through a bottleneck MLP (Equation 1),

$$U=\mathrm{MLP}([Z_{t};Z_{s}])\in R^{B\times C\times D}.$$ (1)

It then computes channel-wise and feature-wise gates with index-stable Top-K softmax (Equations 2 and 3), which retains exactly k entries even when scores are tied. Let $\bar{u}=\frac{1}{C}\sum_{c=1}^{C}U_{\cdot ,c,\cdot }$ denote the mean of U over the C channels. The channel projection $W_{c}$, with shape D×1, maps each channel embedding to a scalar score: ?>

$$g_{c}=C\cdot \mathrm{TopKSoftmax}(UW_{c},k_{c})\in R^{B\times C},$$ (2)

$$g_{d}=D\cdot \mathrm{TopKSoftmax}\;(\mathrm{MLP}(\bar{u}),k_{d})\in R^{B\times D}.$$ (3)

The outer product of the two gates defines a sample-specific correction mask. BM-TopK applies this mask to the spatial representation and adds the result to the temporal residual (Equation 4):

$$Z_{f}=Z_{t}+(g_{c}⊗g_{d})⊙Z_{s}.$$ (4)

The mixing MLP maps 2D→D/4→D. A sparsity ratio r sets $k_{c}=\lfloor Cr\rfloor$ and $k_{d}=\lfloor Dr\rfloor$, with each value constrained to at least one; absolute values of $k_{c}$ and $k_{d}$ may also be specified. Multiplication by C and D preserves the expected gate scale after sparse softmax. Unlike concatenation or dense attention, BM-TopK restricts each sample’s spatial update to selected channels and latent features (Figure 2). BM-TopK therefore provides a sparse injection mechanism. On HC-D, the default setting retains 12 of 48 channels and 32 of 128 latent features per sample; these selected coordinates are reported as model-internal diagnostics rather than as direct physiological markers.

**Figure 2:**
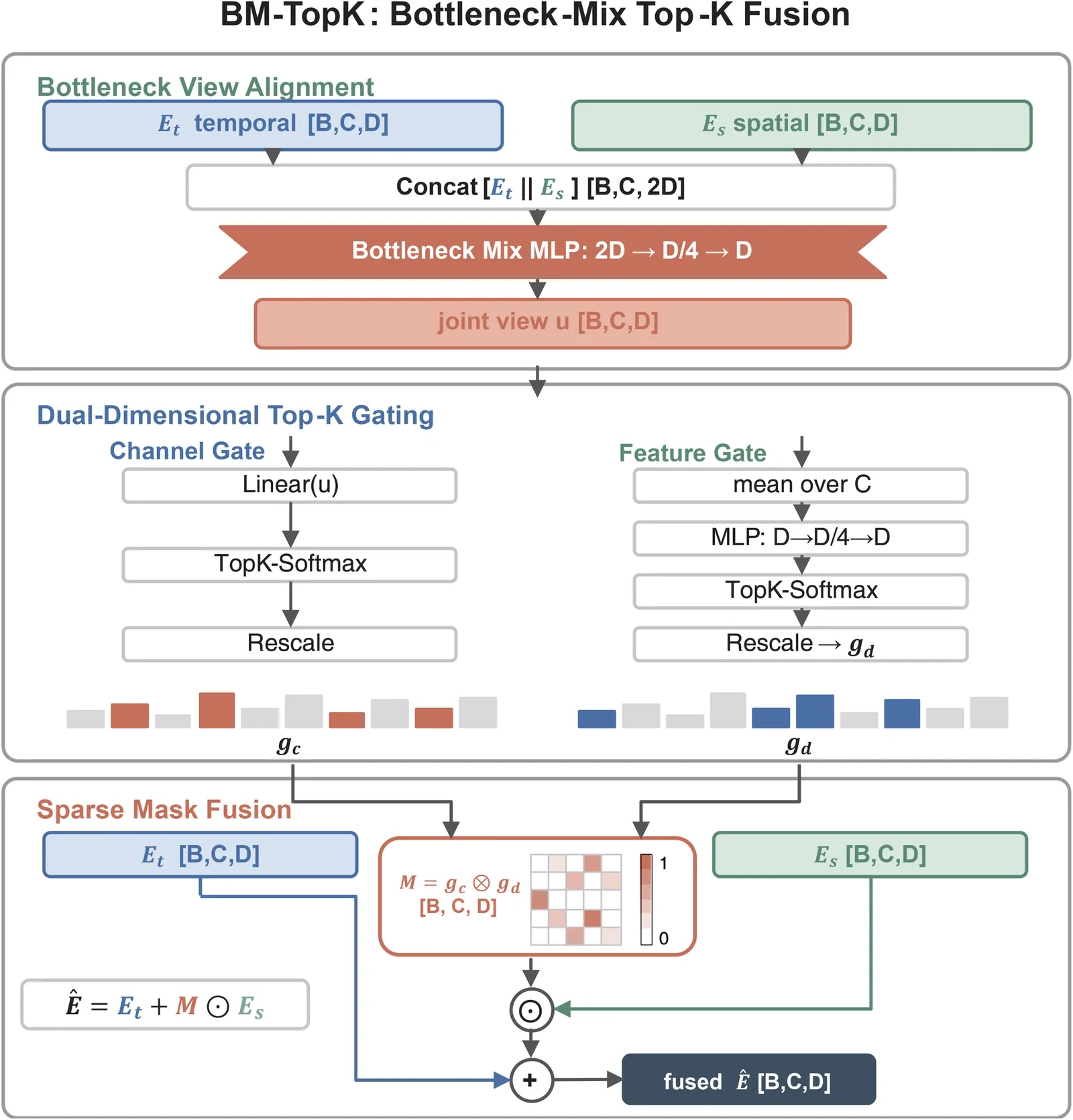
Bottleneck-mix Top-K (BM-TopK) fusion gate. Temporal and spatial embeddings are bottleneck-mixed for dual-axis Top-K scoring; sparse channel and feature gates modulate residual spatial injection into the frozen temporal stream.

After fusion, $Z_{f}$ is pooled over channels and passed to a lightweight prediction head comprising LayerNorm, dropout, and a linear map (Equation 5):

$$z=\mathrm{Dropout}(\mathrm{LayerNorm}(\mathrm{Pool}(Z_{f}))),\;\hat{y}=\mathrm{softmax}(Wz+b).$$ (5)

The model is optimized with cross-entropy loss.

## 4 Experimental setup

### 4.1 Datasets and protocol

We evaluated FroHyNet on five binary classification datasets spanning two imaging modalities (Table 2): HC-D and Yoga-Asana (33) for fNIRS, and Mātai (34), Neurocon, and Taowu (35) for fMRI. Every method was evaluated using stratified five-fold group cross-validation with participant identity as the grouping variable and the fixed seed 42, preventing recordings from the same participant from appearing in both training and test folds. We report accuracy (ACC), area under the receiver-operating-characteristic curve (AUC), precision, recall, F1 score, and the number of trainable parameters. Precision, recall, and F1 are macro-averaged. Accuracy is evaluated at a fixed threshold of 0.5 on predicted probabilities. Fold-level metrics are summarized as mean ± standard deviation. Per-dataset entries report the mean and standard deviation across the five participant-grouped test folds; five-dataset summaries report the mean and standard deviation of the five dataset-level means.

**Table 2:** Summary of the five evaluation datasets.

Dataset	Modality	Classes	Samples	Signal length	Channels/ROIs
HC-D	fNIRS	2	207	720	48
Yoga-Asana	fNIRS	2	27	1,000	20
Mātai	fMRI	2	60	200	116
Neurocon	fMRI	2	41	137	116
Taowu	fMRI	2	40	239	116

fMRI, functional magnetic resonance imaging; fNIRS, functional near-infrared spectroscopy; ROI, region of interest.

The Channels/ROIs column reports 48 fNIRS channels for HC-D, 20 fNIRS channels for Yoga-Asana after excluding two unused short channels, and 116 Automated Anatomical Labeling (AAL) ROIs for each fMRI dataset.

#### 4.1.1 fNIRS preprocessing & datasets

Table 3 summarizes the preprocessing used for each dataset. fNIRS signals were processed at their native lengths; no temporal resampling was applied. Raw fNIRS signals were processed with MNE-NIRS (36). Intensity measurements were converted to optical density and corrected with Temporal Derivative Distribution Repair (TDDR) (37). A 0–0.1 Hz band-pass filter was applied to reduce physiological and systemic noise. Changes in oxygenated hemoglobin (HbO) and deoxygenated hemoglobin (HbR) concentration were then estimated with the modified Beer–Lambert law (38). The classification models used HbO because it generally provides higher signal-to-noise ratio and task sensitivity than HbR (4, 39).

- *HC-D*: This private cohort contains 207 participants, comprising 105 individuals with depression and 102 healthy controls. Signals were recorded with 48 channels during a verbal fluency task and contain 720 time points. The study was approved by the Human Subjects Protection Committee of West China Hospital (approval no. 2023-2157).
- *Yoga-Asana* (33): This public dataset contains recordings from 27 healthy participants, with 20 fNIRS channels (two unused short channels were excluded) and 1,000 time points per signal. The task distinguishes yoga postures from rest.

**Table 3:** Preprocessing summary.

Dataset	Modality	Sampling	Filtering	Motion correction	Signal/input
HC-D	fNIRS	Native rate retained	0–0.1 Hz band-pass	TDDR	HbO channel series
Yoga-Asana	fNIRS	Native rate retained	0–0.1 Hz band-pass	TDDR	HbO channel series
Mātai	fMRI	TR = 1.529 s; T=200	0.01–0.08/0.10 Hz, zero-phase	Rigid-body; 24-param. + WM/CSF; FD/DVARS	AAL-116 mean; session z-score
Neurocon	fMRI	TR = 3480 ms (1.5 T); T=137	0.01–0.08/0.10 Hz, zero-phase	Rigid-body; 24-param. + WM/CSF; FD/DVARS	AAL-116 mean; T=137
Taowu	fMRI	TR = 2000 ms (3 T); T=239	0.01–0.08/0.10 Hz, zero-phase	Rigid-body; 24-param. + WM/CSF; FD/DVARS	AAL-116 mean; T=239

aCompCor, anatomical CompCor; CSF, cerebrospinal fluid; DVARS, temporal derivative of RMS variance over voxels; fMRI, functional magnetic resonance imaging; fNIRS, functional near-infrared spectroscopy; FD, framewise displacement; HbO, oxygenated hemoglobin; ICA-AROMA, independent-component analysis based automatic removal of motion artifacts; ROI, region of interest; TDDR, Temporal Derivative Distribution Repair; TR, repetition time; WM, white matter.

Sampling rates were retained at their native values; no temporal resampling was applied. fNIRS signals were processed with MNE-NIRS. On Mātai, resting-state BOLD was rigidly aligned; 24-parameter motion and WM/CSF were regressed, with aCompCor, linear detrending, and ICA-AROMA when applicable; high-motion volumes were censored by FD/DVARS; a zero-phase band-pass at 0.01–0.08/0.10 Hz was applied; and AAL-116 mean series were session-wise z-scored and retained at T=200. Neurocon and Taowu used the same rigid-body alignment, 24-parameter motion and mean WM/CSF regression (with aCompCor when applicable, linear and quadratic trends, and optional ICA-AROMA), FD/DVARS censoring, and in-sequence zero-phase 0.01–0.08/0.10 Hz band-pass; series were retained at T=137 (Neurocon; 1.5 T Siemens Avanto, TR = 3480 ms) and T=239 (Taowu; 3 T Siemens Magnetom Trio, TR = 2000 ms). Pearson functional connectivity was left unthresholded, including the diagonal.

#### 4.1.2 fMRI preprocessing & datasets

All fMRI datasets were converted to region-of-interest (ROI) time series with the Automated Anatomical Labeling (AAL) atlas (40) to obtain 116 parcels per participant; each ROI series is the mean blood-oxygen-level-dependent (BOLD) signal in that parcel. The three public fMRI benchmarks were converted to a unified array format and length-filtered or truncated to a fixed sequence length. No temporal resampling was applied. For graph-based models, functional-connectivity adjacency matrices were constructed from the training split only to prevent leakage, using unthresholded Pearson correlation (including the diagonal) and symmetrically normalized as $D^{-1/2}(A+I)D^{-1/2}$. FroHyNet used the same training-split adjacency for pairwise graph convolution and constructed sample-adaptive hyperedges from the learned node representations; no external anatomical incidence matrix was required. For fNIRS, FroHyNet used channel-level dynamic hypergraph construction from the data and did not require an external anatomical adjacency or incidence matrix.

- *Mātai* (34): This longitudinal contact-sports resting-state fMRI study contains 60 participants classified as mild traumatic brain injury or control. The repetition time (TR) was 1.529 s. BOLD volumes were rigidly aligned; 24-parameter motion and white-matter (WM) / cerebrospinal-fluid (CSF) signals were regressed, with anatomical CompCor (aCompCor), linear detrending, and ICA-AROMA when applicable; high-motion volumes were censored using framewise displacement (FD) and DVARS. A zero-phase band-pass at 0.01–0.08/0.10 Hz was then applied. Mean AAL-116 time series were z-scored within each session and retained at T=200.
- *Neurocon* (35): This Parkinson’s disease dataset contains 41 participants and 137 time points per sequence. Scans were acquired at 1.5 T (Siemens Avanto) with TR = 3480 ms. BOLD volumes were rigidly aligned; 24-parameter motion and mean WM/CSF were regressed, with aCompCor when applicable, linear and quadratic trends, and optional ICA-AROMA; high-motion volumes were censored using FD and DVARS. A zero-phase band-pass at 0.01–0.08/0.10 Hz was applied in sequence with nuisance regression. Mean AAL-116 series were retained at T=137.
- *Taowu* (35): This dataset contains 20 participants with Parkinson’s disease and 20 age-matched controls, with 239 time points per sequence. Scans were acquired at 3 T (Siemens Magnetom Trio) with TR =2000 ms. The same rigid-body alignment, 24-parameter motion and WM/CSF regression (with aCompCor when applicable, linear and quadratic trends, and optional ICA-AROMA), FD/DVARS censoring, and zero-phase 0.01–0.08/0.10 Hz band-pass as Neurocon were applied; mean AAL-116 series were retained at T=239.

### 4.2 Implementation details

FroHyNet and its branch-layout ablations were trained for at most 100 epochs with a five-epoch warm-up, a minimum of 50 epochs, and early stopping with patience 15. We used AdamW (41) with a learning rate of $10^{-4}$, cosine decay, a batch size of 32, and cross-entropy loss. FroHyNet aligned both branches to D=128 and used mean pooling. Its spatial branch used one 64-dimensional hidden layer, a 64-dimensional temporal compressor, GELU activation, graph convolution with γ=0.5, and sample-adaptive hypergraphs constructed from the three nearest nodes. The default BM-TopK sparsity ratio was r=0.25, giving $k_{c}=\lfloor C/4\rfloor$ and $k_{d}=\lfloor D/4\rfloor$; on HC-D this corresponds to 12 of 48 channels and 32 of 128 latent features per sample. A single frozen MantisV2 checkpoint was shared across all five datasets and remained frozen throughout downstream training. Hyperparameters were selected within the training folds only, using validation accuracy first and validation loss as the tie-breaker; no test-fold information was used for tuning. The same selection rule was applied across datasets and baselines. The same length-handling rule was used for MantisV2, the baselines, and the branch-layout ablations: fNIRS signals were processed at their native lengths, whereas the public fMRI time series were converted to a unified array format and length-filtered or truncated to a fixed sequence length (Mātai retained T=200; Neurocon T=137; Taowu T=239), with no temporal resampling.

### 4.3 Comparison methods

We group the comparison methods by modeling assumption: handcrafted features, from-scratch temporal models, frozen foundation encoders, domain-specific brain time-series models, and graph or spatio-temporal networks. Methods evaluated on only a subset of datasets report $n_{\mathrm{ds}}$. Their averages include only the reported datasets; missing results are not imputed. All methods used the same stratified five-fold participant-grouped splits, with participant identity as the grouping variable, on every dataset they cover. Baselines used published defaults or a small predeclared search restricted to architecture-critical options such as capacity, dropout, and learning rate. Model selection used validation accuracy followed by validation loss within the training folds only; no test-fold information was used for tuning. The same early-stopping and validation-loss tie-breaking rules were applied across method families.

FroHyNet combines frozen MantisV2 (14), a trainable ST-HGNN spatial branch, and BM-TopK fusion. Its 0.118M trainable-parameter count excludes the frozen backbone, which still contributes to storage and inference cost. To isolate the architectural choices, we compare temporal-only, spatial-only, serial, and full parallel layouts on all five datasets. We also compare concatenation, adaptive, LSTM, attention, TAR, and BM-TopK fusion on HC-D using the same temporal and spatial branches.

## 5 Results and discussion

### 5.1 Cross-dataset performance

Figure 3 and Tables 4, 5 summarize the 18 methods evaluated on all five datasets. Per-dataset ACC ranks and their means are calculated only within this full-coverage set. Handcrafted and fNIRS-specific baselines cover only the two fNIRS datasets ($n_{\mathrm{ds}}=2$); their per-dataset results are included in (Tables 5a,b), their fNIRS means are reported separately in Table 6, and they are excluded from the full-coverage averages to avoid comparing averages computed over different datasets.

**Figure 3:**
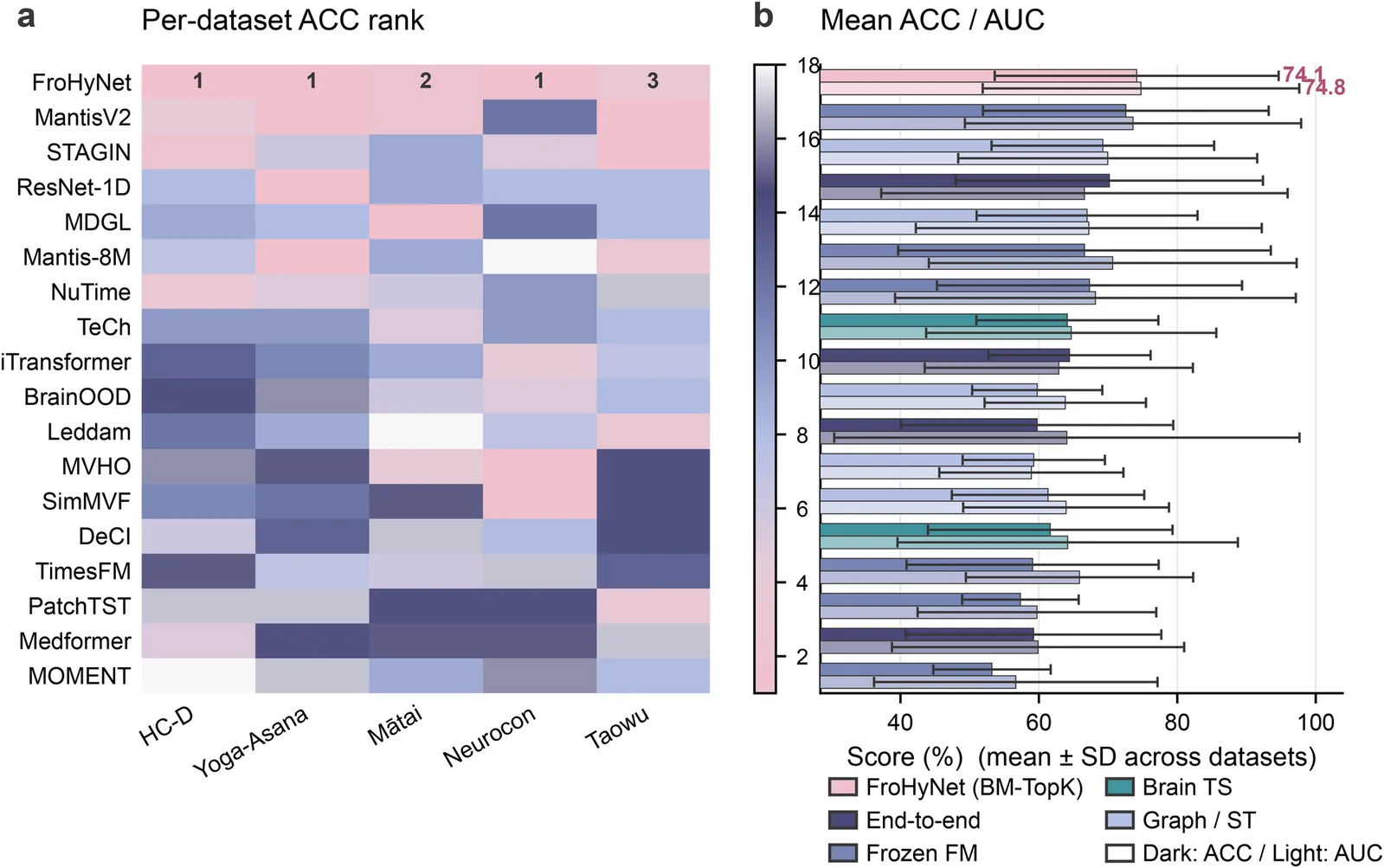
Full-coverage comparison on five datasets. Accuracy (ACC) and area under the receiver-operating-characteristic curve (AUC) are shown. **(a)** Per-dataset ACC ranks (lower/lighter is better). **(b)** Mean ACC (dark) and AUC (light). Bars and error bars show the mean and standard deviation (SD) of the five dataset-level means, matching Table 4. Axis labels and legend entries are enlarged. FroHyNet: 74.13% ACC / 74.75% AUC (rank 1.60).

**Table 4:** Five-dataset means for full-coverage methods (%; Param. in M).

Method	ACC↑	Prec.↑	Rec.↑	F1↑	AUC↑	Rank↓	Param.
*End-to-end*							
ResNet-1D	70.18 ± 22.19	54.07 ± 37.01	67.52 ± 24.39	58.52 ± 32.44	66.58 ± 29.36	6.80	3.910
iTransformer	64.42 ± 11.72	65.21 ± 11.28	61.91 ± 13.13	59.79 ± 12.98	62.88 ± 19.37	8.80	0.655
Medformer	59.24 ± 18.45	56.12 ± 22.64	57.45 ± 19.39	53.92 ± 21.98	59.88 ± 21.11	13.20	2.584
Leddam	59.75 ± 19.66	55.41 ± 25.49	56.85 ± 21.30	53.11 ± 21.76	64.04 ± 33.61	9.80	0.236
*Frozen FM*							
MantisV2	72.56 ± 20.64	**65.74** ± **27.71**	70.62 ± 22.86	**66.85** ± **26.34**	73.60 ± 24.29	4.00	0.165
NuTime	67.31 ± 22.04	59.42 ± 30.40	64.45 ± 24.07	59.58 ± 28.31	68.17 ± 28.95	8.20	0.067
Mantis-8M	66.59 ± 26.92	63.17 ± 30.35	66.10 ± 27.17	62.57 ± 30.41	70.67 ± 26.57	7.60	0.100
TimesFM	59.09 ± 18.21	51.26 ± 26.97	57.39 ± 18.70	51.65 ± 23.21	65.87 ± 16.42	11.60	0.362
PatchTST	57.33 ± 8.42	48.75 ± 16.18	54.74 ± 11.26	49.44 ± 13.84	59.72 ± 17.24	13.00	0.067
MOMENT	53.22 ± 8.48	39.20 ± 21.87	53.93 ± 6.60	41.17 ± 13.02	56.66 ± 20.48	13.60	0.296
*Brain TS*							
TeCh	64.11 ± 13.15	54.81 ± 22.80	60.68 ± 15.82	55.41 ± 20.51	64.67 ± 20.96	8.60	6.070
DeCI	61.64 ± 17.67	54.50 ± 25.77	58.69 ± 19.55	54.11 ± 22.86	64.16 ± 24.59	11.60	0.116
*Graph/ST*							
STAGIN	69.24 ± 16.08	59.39 ± 26.93	65.58 ± 19.24	60.75 ± 23.59	69.94 ± 21.60	4.60	0.349
MDGL	66.96 ± 15.96	54.84 ± 29.56	63.47 ± 18.52	55.79 ± 25.42	67.21 ± 24.99	7.60	0.349
SimMVF	61.32 ± 13.90	51.27 ± 22.56	58.04 ± 15.65	52.08 ± 18.65	63.93 ± 14.87	10.60	0.050
BrainOOD	59.76 ± 9.40	44.67 ± 19.34	57.09 ± 10.36	47.49 ± 15.81	63.81 ± 11.66	9.80	0.028
MVHO	59.26 ± 10.28	44.88 ± 19.01	55.44 ± 11.14	47.70 ± 15.69	58.91 ± 13.30	10.00	0.050
*Proposed*							
**FroHyNet**	**74.13** ± **20.51**	63.92 ± 30.36	**70.79** ± **23.46**	65.69 ± 28.25	**74.75** ± **22.87**	**1.60**	0.118

Values are mean ± SD of the five dataset-level means. Precision, recall, and F1 are macro-averaged; accuracy is evaluated at a decision threshold of 0.5. All tied best values in each column are bolded. Partial-coverage baselines are reported in Table 6.

**Table 5:** Per-dataset results (five-fold mean %).

(a) HC-D					
Method	ACC↑	Prec.↑	Rec.↑	F1↑	AUC↑
ResNet-1D	87.55 ± 5.11	88.69 ± 3.86	87.60 ± 5.09	87.40 ± 5.33	96.44 ± 2.20
iTransformer	77.96 ± 5.88	79.22 ± 5.11	78.05 ± 5.77	77.68 ± 6.13	80.63 ± 6.61
Medformer	89.48 ± 3.55	89.67 ± 3.35	89.48 ± 3.55	89.46 ± 3.58	93.96 ± 1.55
Leddam	78.47 ± 1.52	83.24 ± 2.09	78.57 ± 1.51	77.70 ± 1.88	92.97 ± 5.05
MantisV2	89.49 ± 4.39	89.65 ± 4.34	89.50 ± 4.40	89.48 ± 4.40	96.56 ± 1.27
NuTime	89.95 ± 3.80	90.24 ± 3.55	89.95 ± 3.80	89.93 ± 3.83	96.29 ± 1.82
Mantis-8M	88.52 ± 4.08	88.68 ± 3.90	88.52 ± 4.08	88.51 ± 4.10	95.64 ± 1.53
TimesFM	74.16 ± 8.31	75.03 ± 7.76	74.19 ± 8.31	73.69 ± 8.98	84.44 ± 5.13
PatchTST	72.23 ± 4.28	73.88 ± 4.10	72.26 ± 4.25	71.72 ± 4.52	79.95 ± 3.05
MOMENT	65.01 ± 8.82	72.67 ± 6.57	65.24 ± 8.46	61.27 ± 13.29	78.78 ± 7.60
TeCh	79.88 ± 3.69	80.46 ± 3.29	79.95 ± 3.62	79.79 ± 3.78	89.19 ± 3.31
DeCI	89.00 ± 3.85	89.18 ± 3.83	89.02 ± 3.86	88.99 ± 3.86	94.78 ± 2.85
STAGIN	90.43 ± 2.61	91.12 ± 2.16	90.43 ± 2.61	90.38 ± 2.65	93.66 ± 2.24
MDGL	87.06 ± 4.49	88.11 ± 3.90	87.10 ± 4.46	86.95 ± 4.58	94.98 ± 1.57
SimMVF	78.92 ± 4.26	79.26 ± 4.27	78.90 ± 4.28	78.85 ± 4.30	80.61 ± 4.60
BrainOOD	75.08 ± 6.00	77.09 ± 5.64	75.00 ± 6.12	74.45 ± 6.40	82.21 ± 8.87
MVHO	73.19 ± 2.88	73.59 ± 3.50	73.21 ± 2.87	73.13 ± 2.77	73.80 ± 5.16
Handcraft+MLP	87.08 ± 3.86	87.47 ± 3.94	87.12 ± 3.86	87.06 ± 3.85	94.21 ± 3.24
Handcraft+GCN	85.66 ± 2.97	86.31 ± 2.94	85.69 ± 2.99	85.59 ± 3.00	94.93 ± 1.84
MaNDiNeT	80.85 ± 1.68	82.57 ± 1.69	80.93 ± 1.54	80.62 ± 1.77	90.17 ± 1.61
fNIRS-T	73.66 ± 4.68	73.77 ± 4.68	73.64 ± 4.70	73.61 ± 4.70	83.92 ± 5.27
LoBrAFrame	75.56 ± 4.35	76.77 ± 3.45	75.64 ± 4.21	75.25 ± 4.63	82.70 ± 3.03
fNIRS-PreT	68.86 ± 6.07	70.86 ± 5.95	68.95 ± 5.95	68.07 ± 6.37	78.15 ± 10.09
**FroHyNet**	**92.33** ± **2.81**	**92.51** ± **2.76**	**92.36** ± **2.79**	**92.33** ± **2.81**	**97.56** ± **0.64**

(b) Yoga-Asana					
Method	ACC↑	Prec.↑	Rec.↑	F1↑	AUC↑
ResNet-1D	**100.00** ± **0.00**	**100.00** ± **0.00**	**100.00** ± **0.00**	**100.00** ± **0.00**	**100.00** ± **0.00**
iTransformer	75.56 ± 16.33	75.60 ± 24.94	74.40 ± 18.26	69.60 ± 22.76	87.00 ± 21.35
Medformer	63.11 ± 16.37	67.60 ± 19.40	62.00 ± 15.22	60.69 ± 16.05	60.80 ± 20.80
Leddam	76.67 ± 17.36	76.40 ± 26.45	75.40 ± 18.13	71.14 ± 23.46	98.75 ± 2.17
MantisV2	**100.00** ± **0.00**	**100.00** ± **0.00**	**100.00** ± **0.00**	**100.00** ± **0.00**	**100.00** ± **0.00**
NuTime	91.33 ± 8.27	93.60 ± 5.57	91.00 ± 9.17	90.76 ± 9.20	**100.00** ± **0.00**
Mantis-8M	**100.00** ± **0.00**	**100.00** ± **0.00**	**100.00** ± **0.00**	**100.00** ± **0.00**	**100.00** ± **0.00**
TimesFM	80.44 ± 10.83	84.40 ± 10.52	80.00 ± 11.40	79.32 ± 11.46	80.40 ± 16.08
PatchTST	54.44 ± 12.37	45.80 ± 23.22	54.00 ± 11.12	46.13 ± 15.93	72.80 ± 10.07
MOMENT	54.44 ± 7.37	50.00 ± 21.11	54.40 ± 8.69	46.81 ± 13.00	75.00 ± 17.61
TeCh	76.22 ± 12.56	78.14 ± 12.91	75.50 ± 12.69	75.26 ± 12.83	80.60 ± 21.39
DeCI	67.56 ± 13.45	74.00 ± 13.96	67.60 ± 12.21	65.33 ± 14.23	87.00 ± 12.88
STAGIN	82.44 ± 9.06	85.40 ± 9.46	81.60 ± 10.01	81.41 ± 9.88	92.00 ± 9.27
MDGL	80.22 ± 8.56	82.60 ± 9.16	79.60 ± 9.52	79.24 ± 9.30	91.20 ± 5.98
SimMVF	69.33 ± 16.59	72.20 ± 25.69	70.60 ± 13.76	64.65 ± 21.17	78.60 ± 14.55
BrainOOD	56.22 ± 10.74	41.94 ± 22.02	57.00 ± 8.72	46.38 ± 17.26	67.00 ± 17.78
MVHO	56.44 ± 10.10	55.00 ± 19.70	59.00 ± 9.25	52.79 ± 14.16	63.20 ± 17.92
Handcraft+MLP	82.44 ± 9.06	84.67 ± 9.51	82.50 ± 9.49	81.91 ± 9.60	92.00 ± 6.78
Handcraft+GCN	63.11 ± 8.39	61.49 ± 9.43	61.49 ± 9.43	61.49 ± 9.43	65.80 ± 9.93
MaNDiNeT	83.33 ± 21.08	83.33 ± 21.08	83.00 ± 21.35	82.00 ± 22.93	86.20 ± 17.94
fNIRS-T	71.56 ± 9.26	69.99 ± 11.30	69.99 ± 11.30	69.99 ± 11.30	80.20 ± 12.50
LoBrAFrame	67.78 ± 20.00	70.75 ± 20.52	67.00 ± 19.96	65.98 ± 20.41	72.20 ± 21.25
fNIRS-PreT	67.11 ± 10.46	65.49 ± 11.87	65.49 ± 11.87	65.49 ± 11.87	76.00 ± 3.74
**FroHyNet**	**100.00** ± **0.00**	**100.00** ± **0.00**	**100.00** ± **0.00**	**100.00** ± **0.00**	**100.00** ± **0.00**

(c) Mātai					
Method	ACC↑	Prec.↑	Rec.↑	F1↑	AUC↑
ResNet-1D	55.00 ± 6.67	27.50 ± 3.33	50.00 ± 0.00	35.36 ± 2.97	37.14 ± 7.45
iTransformer	55.00 ± 17.16	55.25 ± 20.72	52.29 ± 16.08	51.59 ± 16.66	46.29 ± 24.07
Medformer	50.00 ± 19.00	47.43 ± 25.86	46.29 ± 18.26	43.11 ± 18.94	49.14 ± 12.31
Leddam	30.00 ± 8.50	22.34 ± 5.79	26.29 ± 7.59	24.03 ± 6.26	15.43 ± 12.73
MantisV2	60.00 ± 3.33	**55.38** ± **13.63**	**59.43** ± **5.97**	**55.53** ± **9.92**	**70.29** ± **7.58**
NuTime	56.67 ± 6.24	48.43 ± 18.34	53.14 ± 6.09	44.74 ± 9.40	63.43 ± 13.08
Mantis-8M	55.00 ± 11.30	50.76 ± 18.59	52.29 ± 9.24	48.32 ± 11.53	54.29 ± 10.06
TimesFM	56.67 ± 3.33	28.79 ± 0.76	48.57 ± 2.86	36.14 ± 1.40	50.86 ± 16.00
PatchTST	53.33 ± 16.33	45.12 ± 20.94	51.43 ± 13.58	45.95 ± 17.53	54.86 ± 22.16
MOMENT	55.00 ± 6.67	27.50 ± 3.33	50.00 ± 0.00	35.36 ± 2.97	42.29 ± 17.09
TeCh	58.33 ± 5.27	44.89 ± 14.64	52.29 ± 5.76	45.90 ± 9.57	37.14 ± 24.04
DeCI	48.33 ± 9.72	33.33 ± 9.03	43.14 ± 8.45	37.01 ± 7.46	44.57 ± 25.97
STAGIN	55.00 ± 4.08	33.58 ± 10.75	47.71 ± 4.10	37.99 ± 6.01	56.57 ± 9.46
MDGL	**61.67** ± **4.08**	50.23 ± 25.79	54.00 ± 4.90	44.33 ± 9.17	62.86 ± 15.86
SimMVF	50.00 ± 7.45	36.29 ± 11.95	45.71 ± 6.76	39.28 ± 9.03	54.29 ± 16.66
BrainOOD	56.67 ± 8.16	39.58 ± 14.67	51.43 ± 6.13	42.86 ± 9.97	58.29 ± 18.12
MVHO	58.33 ± 0.00	29.17 ± 0.00	50.00 ± 0.00	36.84 ± 0.00	58.29 ± 14.64
**FroHyNet**	60.00 ± 8.16	48.78 ± 17.56	56.57 ± 9.71	49.33 ± 13.87	66.29 ± 9.96

(d) Neurocon					
Method	ACC↑	Prec.↑	Rec.↑	F1↑	AUC↑
ResNet-1D	58.33 ± 10.54	29.17 ± 5.27	50.00 ± 0.00	36.53 ± 4.67	49.33 ± 29.09
iTransformer	61.11 ± 13.97	**57.20** ± **25.84**	**52.33** ± **14.44**	**50.54** ± **16.22**	48.00 ± 18.57
Medformer	51.11 ± 13.87	43.29 ± 19.97	47.00 ± 15.51	40.55 ± 13.74	58.00 ± 21.46
Leddam	58.61 ± 12.28	38.42 ± 17.63	49.00 ± 14.20	42.65 ± 15.88	55.56 ± 26.37
MantisV2	55.83 ± 15.50	35.67 ± 14.21	46.67 ± 14.30	39.77 ± 13.30	44.89 ± 13.58
NuTime	56.11 ± 9.69	32.50 ± 2.50	45.67 ± 5.54	37.52 ± 1.18	41.11 ± 6.59
Mantis-8M	34.44 ± 14.99	26.42 ± 9.91	34.67 ± 10.87	27.63 ± 8.37	38.44 ± 11.45
TimesFM	36.67 ± 11.30	25.43 ± 4.46	36.67 ± 8.94	28.33 ± 4.08	48.67 ± 27.29
PatchTST	51.67 ± 10.74	28.93 ± 2.30	41.00 ± 9.17	33.72 ± 4.93	36.00 ± 25.16
MOMENT	41.67 ± 10.54	20.83 ± 5.27	50.00 ± 0.00	29.06 ± 4.78	56.00 ± 16.11
TeCh	56.11 ± 9.69	32.50 ± 2.50	45.67 ± 5.54	37.52 ± 1.18	52.67 ± 12.72
DeCI	58.33 ± 13.18	44.39 ± 21.83	48.67 ± 11.66	43.81 ± 13.02	48.22 ± 19.27
STAGIN	60.83 ± 12.53	38.83 ± 17.47	50.67 ± 13.73	43.51 ± 15.60	46.22 ± 20.24
MDGL	55.83 ± 15.50	28.27 ± 7.04	46.67 ± 6.67	35.08 ± 7.56	45.78 ± 23.92
SimMVF	**63.33** ± **1.67**	31.67 ± 0.83	50.00 ± 0.00	38.77 ± 0.62	58.67 ± 15.43
BrainOOD	60.83 ± 5.65	39.70 ± 15.88	52.00 ± 4.00	40.41 ± 3.18	51.56 ± 11.71
MVHO	**63.33** ± **1.67**	31.67 ± 0.83	50.00 ± 0.00	38.77 ± 0.62	**61.78** ± **17.74**
**FroHyNet**	**63.33** ± **1.67**	31.67 ± 0.83	50.00 ± 0.00	38.77 ± 0.62	48.67 ± 22.67

(e) Taowu					
Method	ACC↑	Prec.↑	Rec.↑	F1↑	AUC↑
ResNet-1D	50.00 ± 0.00	25.00 ± 0.00	50.00 ± 0.00	33.33 ± 0.00	50.00 ± 27.95
iTransformer	52.50 ± 12.25	**58.76** ± **18.88**	52.50 ± 12.25	49.53 ± 10.82	52.50 ± 20.39
Medformer	42.50 ± 12.75	32.62 ± 16.72	42.50 ± 12.75	35.80 ± 14.22	37.50 ± 9.68
Leddam	55.00 ± 12.75	56.67 ± 22.46	55.00 ± 12.75	50.06 ± 14.99	57.50 ± 26.63
MantisV2	**57.50** ± **21.79**	48.00 ± 31.79	**57.50** ± **21.79**	49.46 ± 26.04	56.25 ± 25.92
NuTime	42.50 ± 10.00	32.33 ± 9.92	42.50 ± 10.00	34.97 ± 6.98	40.00 ± 20.77
Mantis-8M	55.00 ± 12.75	50.00 ± 24.01	55.00 ± 12.75	48.39 ± 17.01	**65.00** ± **20.00**
TimesFM	47.50 ± 14.58	42.67 ± 23.63	47.50 ± 14.58	40.77 ± 15.34	**65.00** ± **14.03**
PatchTST	55.00 ± 18.71	50.00 ± 26.35	55.00 ± 18.71	49.67 ± 21.72	55.00 ± 28.61
MOMENT	50.00 ± 0.00	25.00 ± 0.00	50.00 ± 0.00	33.33 ± 0.00	31.25 ± 10.46
TeCh	50.00 ± 7.91	38.05 ± 20.76	50.00 ± 7.91	38.57 ± 8.98	63.75 ± 20.31
DeCI	45.00 ± 6.12	31.62 ± 10.53	45.00 ± 6.12	35.42 ± 6.37	46.25 ± 18.79
STAGIN	**57.50** ± **16.96**	48.00 ± 25.24	**57.50** ± **16.96**	**50.48** ± **21.31**	61.25 ± 15.51
MDGL	50.00 ± 0.00	25.00 ± 0.00	50.00 ± 0.00	33.33 ± 0.00	41.25 ± 15.61
SimMVF	45.00 ± 12.75	36.95 ± 16.69	45.00 ± 12.75	38.84 ± 13.78	47.50 ± 25.80
BrainOOD	50.00 ± 0.00	25.00 ± 0.00	50.00 ± 0.00	33.33 ± 0.00	60.00 ± 31.02
MVHO	45.00 ± 10.00	35.00 ± 12.25	45.00 ± 10.00	37.00 ± 8.46	37.50 ± 15.81
**FroHyNet**	55.00 ± 18.71	46.67 ± 26.67	55.00 ± 18.71	48.00 ± 21.59	61.25 ± 26.34

Entries are mean ± SD across five participant-grouped test folds. Precision, recall, and F1 are macro-averaged; accuracy is evaluated at a decision threshold of 0.5. All tied best values in each column are bolded. Subtables **(a)** and **(b)** additionally include the partial-coverage baselines evaluated only on the two fNIRS datasets ($n_{\mathrm{ds}}=2$; below the horizontal rule).

**Table 6:** Two-dataset fNIRS means for partial-coverage baselines ($n_{\mathrm{ds}}=2$: HC-D and Yoga-Asana; %; Param. in M).

Method	ACC↑	Prec.↑	Rec.↑	F1↑	AUC↑	Param.
*Handcrafted*						
Handcraft+MLP	84.76 ± 3.28	86.07 ± 1.98	84.81 ± 3.27	84.48 ± 3.64	93.10 ± 1.56	0.038
Handcraft+GCN	74.38 ± 15.95	73.90 ± 17.55	73.59 ± 17.11	73.54 ± 17.04	80.36 ± 20.60	0.021
*fNIRS-specific*						
MaNDiNeT	82.09 ± 1.75	82.95 ± 0.54	81.96 ± 1.46	81.31 ± 0.98	88.19 ± 2.81	0.404
fNIRS-T	72.61 ± 1.48	71.88 ± 2.67	71.81 ± 2.58	71.80 ± 2.56	82.06 ± 2.63	1.899
LoBrAFrame	71.67 ± 5.50	73.76 ± 4.26	71.32 ± 6.11	70.62 ± 6.55	77.45 ± 7.42	0.073
fNIRS-PreT	67.99 ± 1.24	68.17 ± 3.80	67.22 ± 2.45	66.78 ± 1.82	77.07 ± 1.52	1.903
*Proposed (fNIRS mean, reference)*						
**FroHyNet**	**96.17** ± **5.42**	**96.26** ± **5.30**	**96.18** ± **5.40**	**96.16** ± **5.42**	**98.78** ± **1.73**	**0.118**

Values are mean ± SD of the two dataset-level means. Precision, recall, and F1 are macro-averaged; accuracy is evaluated at a decision threshold of 0.5. All tied best values in each column are bolded. FroHyNet fNIRS means shown for reference; not directly comparable with the five-dataset means in Table 4.

Among methods evaluated on all five datasets, FroHyNet records the highest mean ACC (74.13%), the highest mean AUC (74.75%), and the lowest mean rank (1.60) (Table 4). MantisV2 reaches 72.56% ACC with a mean rank of 4.00, and STAGIN reaches 69.24% with a mean rank of 4.60. The aggregate gain is driven mainly by the two fNIRS datasets: FroHyNet reaches 96.17% mean ACC on fNIRS, whereas performance on the three smaller fMRI cohorts is more modest (59.44% mean ACC), compared with 57.78% for both MantisV2 and STAGIN.

On the two fNIRS datasets, the partial-coverage baselines provide a further reference point (Table 6). The strongest is Handcraft+MLP (84.76% ACC, 93.10% AUC), followed by MaNDiNeT (82.09% ACC); fNIRS-T, LoBrAFrame, and fNIRS-PreT reach 67.99–72.61% ACC. FroHyNet’s fNIRS average (96.17% ACC, 98.78% AUC) remains above all of these baselines, while its trainable-parameter count is lower than those of MaNDiNeT, fNIRS-T, and fNIRS-PreT.

### 5.2 Dataset-level behavior

Performance also varies substantially by dataset. FroHyNet records the highest ACC on HC-D, shares a perfect score with ResNet-1D, MantisV2, and Mantis-8M on Yoga-Asana, and ties SimMVF and MVHO for the highest ACC on Neurocon; it is second on Mātai (level with MantisV2) and shares third place on Taowu (Figure 3; Table 5). Its perfect Yoga-Asana score should be interpreted in light of the dataset’s 27 participants and comparatively simple posture-vs.-rest task; several methods reach perfect accuracy on this saturated cohort. Conversely, ACC remains modest on Mātai (60.00%) and Taowu (55.00%), with substantial fold-to-fold variation. On Neurocon, the 63.33% accuracy coexists with an AUC of 48.67%, reflecting threshold-dependent behavior in a small cohort of 41 participants. The fixed decision threshold can yield favorable accuracy even when the model’s score ordering is close to chance, and fold composition and class prevalence can amplify this dissociation; FroHyNet’s AUC therefore trails MVHO (61.78%) and SimMVF (58.67%). We frame this result as threshold-dependent rather than as stable cross-cohort discrimination. These results limit any claim of uniform cross-dataset superiority.

### 5.3 Parameter efficiency and claim boundary

FroHyNet uses 0.118M trainable parameters (Figure 4), markedly fewer than the from-scratch ResNet-1D, Medformer, and iTransformer, while matching or exceeding their modality-level ACC. This comparison counts parameters optimized on the target cohort, not total model size. BrainOOD and NuTime have smaller trainable heads, and the frozen MantisV2 backbone still contributes to storage and inference. Relative to from-scratch temporal models, FroHyNet is an accuracy–trainable-parameter trade-off, not the smallest model overall.

**Figure 4:**
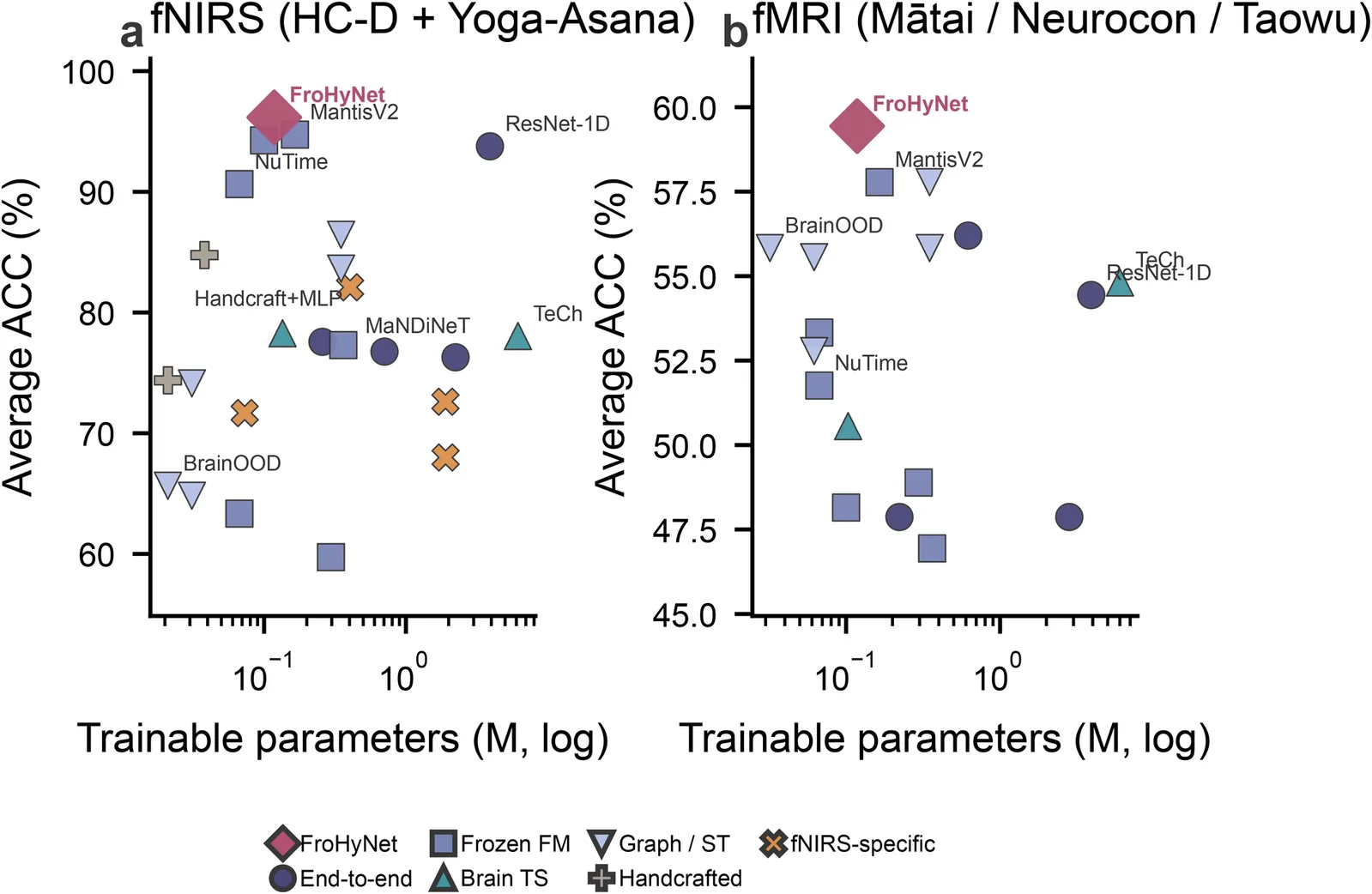
Trainable parameters vs. accuracy (ACC). **(a)** functional near-infrared spectroscopy (fNIRS), including the handcrafted and fNIRS-specific partial-coverage baselines ($n_{\mathrm{ds}}=2$); **(b)** functional magnetic resonance imaging (fMRI). FroHyNet is favorable against from-scratch end-to-end models, but not overall parameter-minimal (BrainOOD and NuTime are smaller).

### 5.4 Ablation studies

The branch ablation shows that the benefit of parallel encoding is dataset-dependent (Figure 5a). The full model is best or tied on HC-D and Yoga-Asana, whereas the temporal-only branch remains competitive on Yoga-Asana and Neurocon. The spatial-only branch performs favorably on Mātai, and the serial layout is weaker except on high-variance Taowu. Thus, the two branches provide complementary information in some cohorts, but the current evidence does not support a universal advantage for the parallel layout.

**Figure 5:**
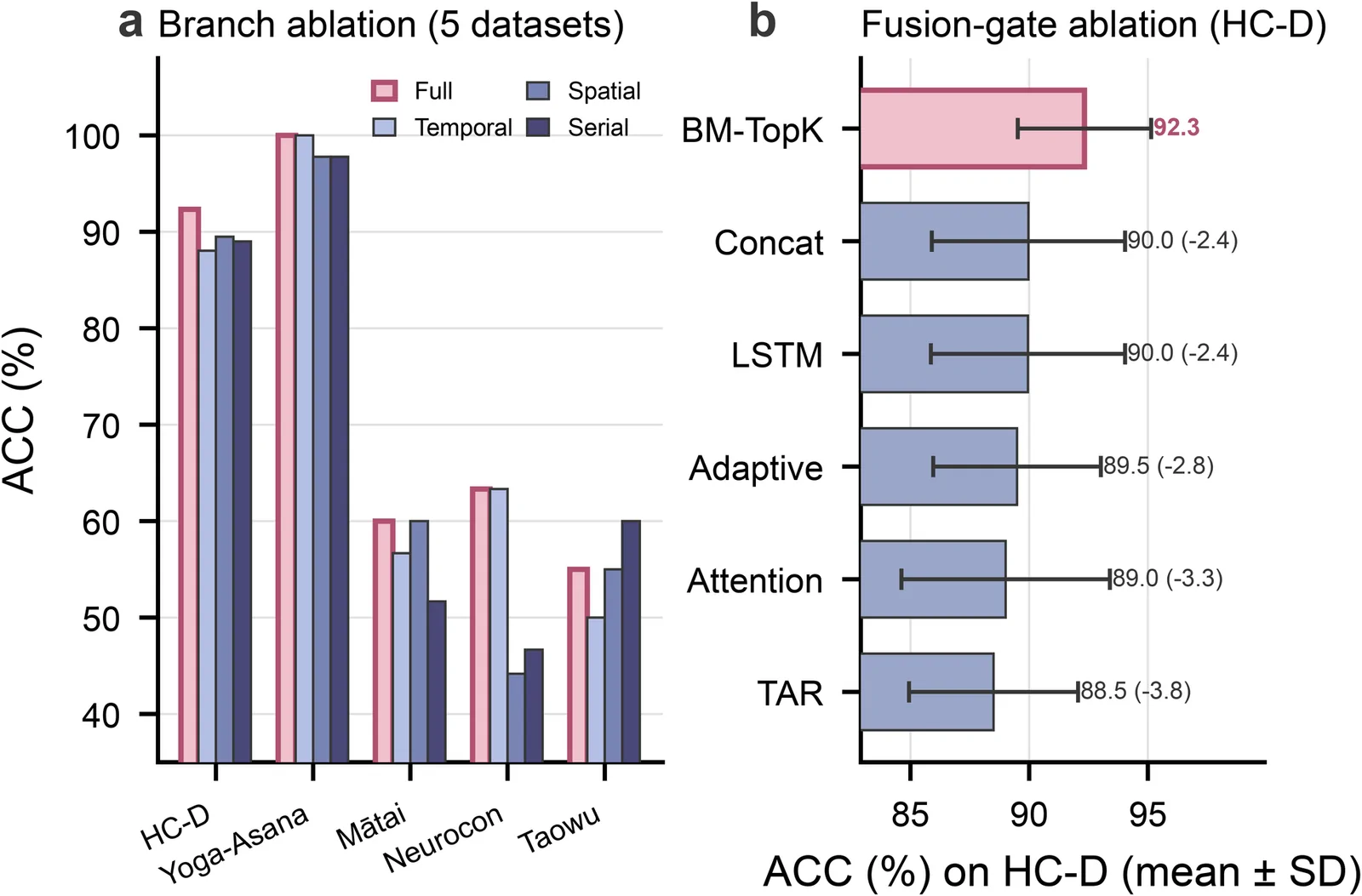
Ablations. **(a)** Branch layouts on five datasets: full parallel vs. temporal-only, spatial-only, and serial MantisV2→HGCN. **(b)** Fusion gates on HC-D: bottleneck-mix Top-K (BM-TopK) vs. attention, concat, long short-term memory (LSTM), adaptive, and token aggregation–redistribution (TAR). HC-D is the private fNIRS depression cohort. Bars in **(b)** show mean accuracy (ACC) ± standard deviation (SD) across five participant-grouped test folds.

The fusion ablation isolates the effect of BM-TopK on HC-D (Figure 5b). BM-TopK reaches 92.33±2.81% ACC and 97.56±0.64% AUC, compared with 89.01±4.39% ACC for attention, approximately 89.97% for concatenation and LSTM, 89.49% for adaptive fusion, and 88.50% for TAR. Under these runs, BM-TopK also obtains the highest AUC and F1 score. The fusion operators have not yet been compared on the fMRI datasets.

To inspect the geometry of these representations, we visualized HC-D test embeddings for the four branch and fusion configurations using t-SNE (42) (Figure 6). The temporal-only MantisV2 embeddings showed moderate overlap between healthy controls (HC) and participants with depression (D); the spatial-only ST-HGNN embeddings produced a clearer diagonal trend but still mixed the two groups; and the serial MantisV2→HGCN layout remained highly intermingled. In contrast, the full FroHyNet (BM-TopK) embeddings separated HC and D samples along the major axis of variation, with most HC points concentrated at lower t-SNE 1 values and D points extending toward higher values. The HC-D embedding geometry matches the branch and fusion accuracies on this cohort.

**Figure 6:**
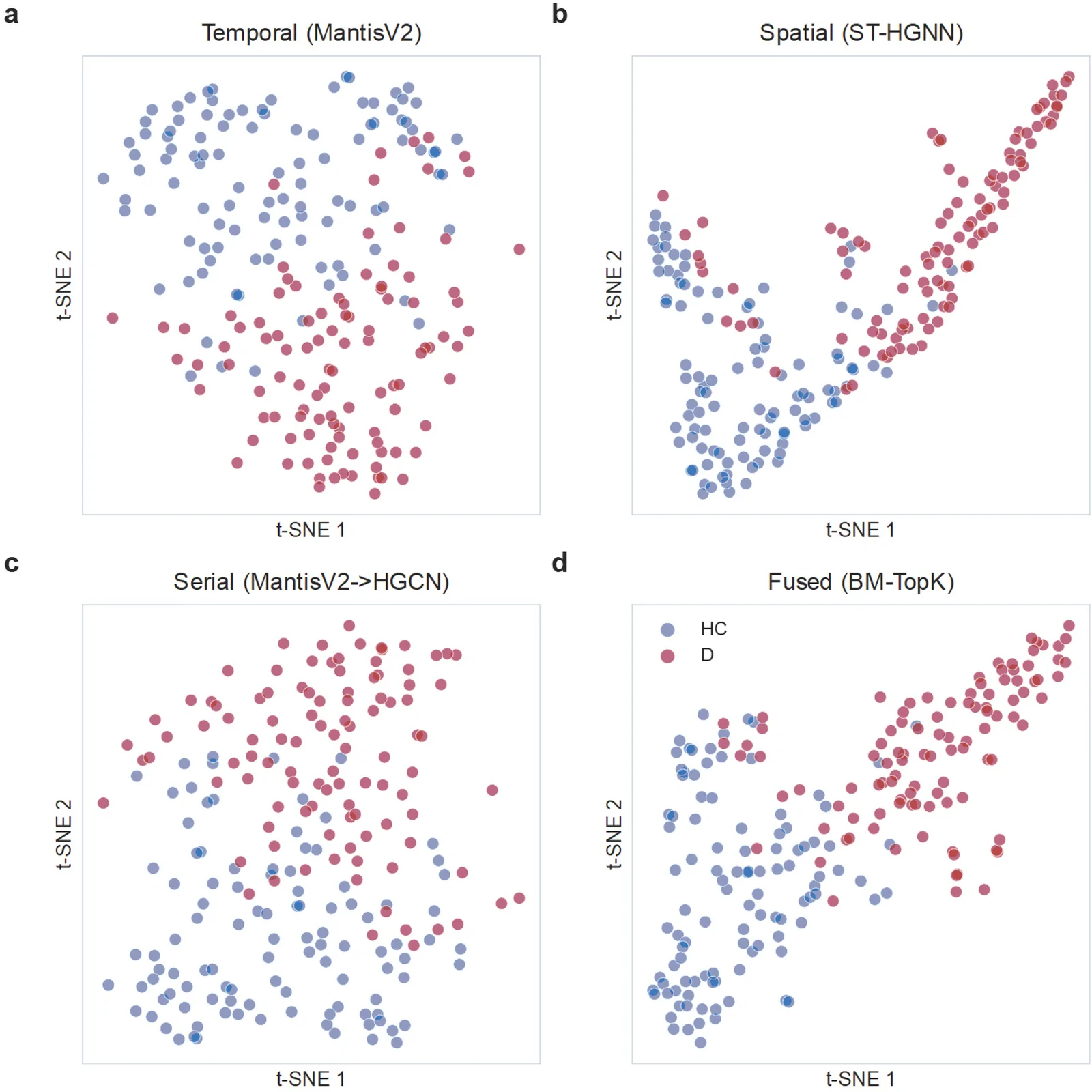
t-distributed stochastic neighbor embedding (t-SNE) visualization of HC-D test embeddings. **(a)** Temporal-only MantisV2. **(b)** Spatial-only ST-HGNN. **(c)** Serial MantisV2→HGCN. **(d)** Full FroHyNet with BM-TopK fusion. HC-D is the private fNIRS depression cohort; HC, healthy controls; D, depression.

## 6 Conclusion

FroHyNet addresses small-cohort brain time-series classification by preserving frozen temporal representations and learning topology-aware spatial corrections in parallel. BM-TopK injects these corrections only along selected channel and feature axes. Across five fNIRS and fMRI datasets, this design achieves a favorable accuracy–trainable-parameter trade-off relative to from-scratch temporal models. FroHyNet reaches 96.17% mean ACC on the two fNIRS datasets and 59.44% mean ACC on the three smaller fMRI datasets. Gains are therefore concentrated on fNIRS, with more modest improvements on the smaller fMRI cohorts. The fusion comparison is currently confined to HC-D.

## Data Availability

The raw data supporting the conclusions of this article will be made available by the authors, without undue reservation.
